# Whole-genome sequencing of novel pathogenic and multidrug-resistant *Kytococcus sedentarius*, causing mortality in fish species (*Labeo rohita*)

**DOI:** 10.1128/mra.01127-24

**Published:** 2024-12-16

**Authors:** Basanta Kumar Das, Vikash Kumar, Suvra Roy, Debasmita Mohanty, Asim Kumar Jana, Ayushman Gadnayak

**Affiliations:** 1 ICAR-Central Inland Fisheries Research Institute, Barrackpore, Kolkata, India; Rochester Institute of Technology, Rochester, New York, USA

**Keywords:** whole genome, pathogenic, multidrug-resistant, *Kytococcus sedentarius*, *Labeo rohita*

## Abstract

We present a novel pathogenic and multidrug-resistant *Kytococcus sedentarius* isolated from *Labeo rohita*. The bacterium belongs to the Micrococcales order and has a genome consisting of 2.59 Mb in length and 71.7% GC content. *K. sedentarius* contains 2,393 coding gene sequences, 6 rRNA, 51 tRNA, 1 tmRNA, and 2,173 antimicrobial resistance genes.

## ANNOUNCEMENT

*Kytococcus sedentarius* (family Kytococcaceae), ubiquitously found in aquatic and terrestrial environments, is characterized as a spherical/coccoid, Gram-positive, non-motile, non-encapsulated, and non-endospore-forming bacterium (1, 2). *K. sedentarius* CP169391 was isolated from *Labeo rohita* cultured in Sardar Bherry located in the East Kolkata Wetland, West Bengal, India. The symptomatic fish tissue samples were homogenized aseptically and transferred to a conical flask containing sterile tryptone soya broth (TSB) (3). Afterward, based on uniqueness in morphology, size, and color, a single colony was picked from TSA plates and cultured overnight in TSB at 28°C. Biochemical assay and 16S rRNA gene sequencing determined by Sanger sequencing before genome sequencing confirmed the presence of the Enterococcaceae family (4). Based on *in vitro* and *in vivo* characterization and an antibiogram assay, the isolate was also confirmed for high virulence and multiple antibiotic resistance (5).

Bacteria were grown in TSA at 28°C under aerobic conditions for 24 hours. The colonies were scraped, suspended in TE buffer, and pelleted by centrifugation. Genomic DNA was extracted from the pellet using a Qiagen DNeasy PowerSoil Pro Kit (Cat. No: 47014). DNA size distribution (Agilent FEMTO pulse, Agilent Technologies, USA), DNA quality check (NanoDrop Spectrophotometer and Qubit3.0 Fluorimeter, ThermoFisher Scientific, USA), DNA shearing (Megaruptor 3, Belgium), sheared DNA and SMRTbell library size distribution (Agilent FEMTO pulse, Agilent Technologies, USA), SMRTbell library preparation, SMRT library purification, library size selection to remove <5 KB fragments, and preparation of bound complex (Pacific Biosciences, USA) were performed for sequencing (supplementary). *De novo* whole-genome sequencing was performed on the PacBio sequel II (Nucleome Informatics Pvt Ltd, Pacific Biosciences, USA). The generated raw subreads were converted to HiFi reads using ccs (version 6.2.0) (6). HiFi reads provide base-level resolution with 99.9% single-molecule read accuracy. For genome assembly and assessment, CCS reads are used to assemble the genomes using the Flyev2.9.3 and *de novo* assembler for single molecule sequencing reads using repeat graphs. Then, genome completeness was done using the tools “QUAST” and “BUSCO” using bacteria_odb10 as lineage (7, 8). The ntCard tool is used for genome surveys (9). At its core, ntCard uses the ntHash algorithm to compute hash values for streamed sequences efficiently. Eggnogmapper is used for functional annotation (10). It finds and annotates features (both protein-coding regions and RNA genes, i.e., tRNA and rRNA) present in a sequence (11). The antimicrobial resistance genes are predicted based on homology and SNP models. The Resistance Gene Identifier tool uses reference data from the Comprehensive Antibiotic Resistance Database (12). A whole genome phylogeny was constructed using CLC genomics workbench 12. The k-mer-based tree was built using a neighbor-joining approach, and Mahalanobis’ methods measured the evolutionary distance.

**Fig 1:**
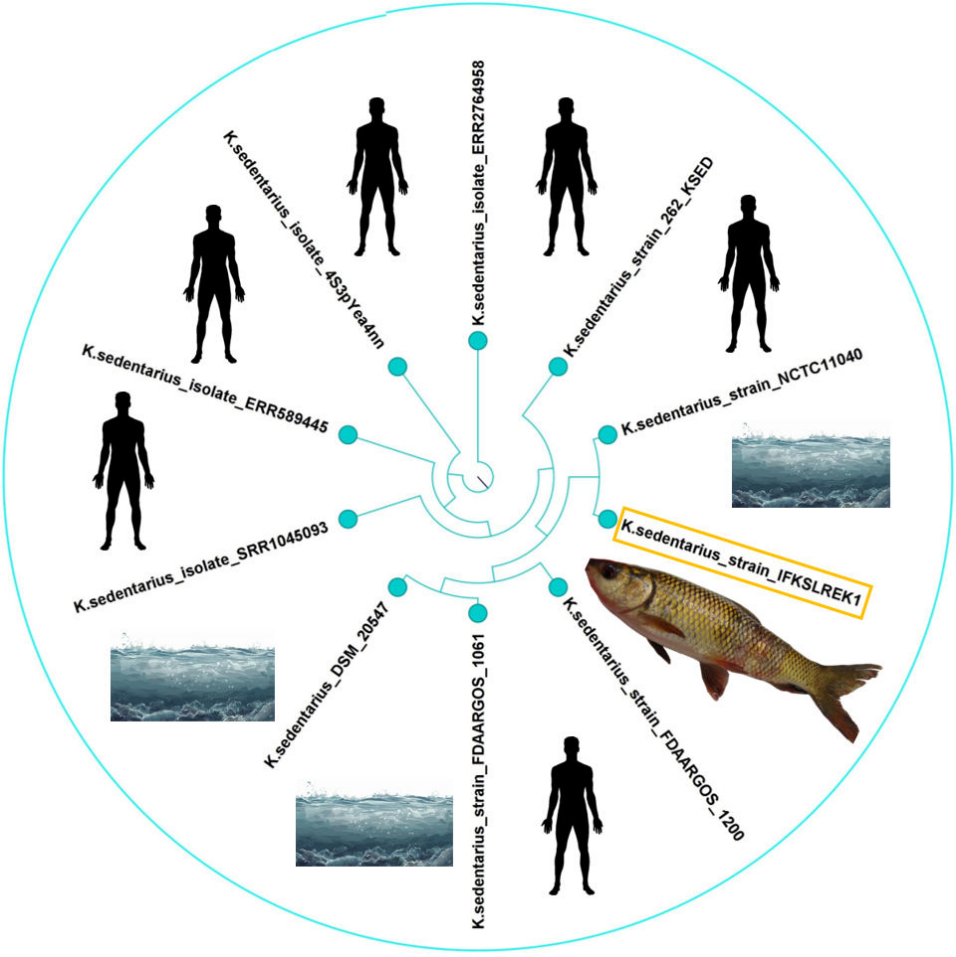
The phylogenetic tree comprises isolated *Kytococcus sedentarius* and strains from different sources and countries. This figure was generated using the CLC genomics workbench, and the k-mer-based tree was built using a neighbor-joining approach and Mahalanobis’ methods.

The genomic assembly features of *K. sedentarius* CP169391 are presented in Table 1. The genome assembled in this study was 97.6% complete and 0.0% contaminated. It consisted of a closed, circular chromosome of 2,598,617 bp, with a G + C content of 71.7%. We identified different antimicrobial resistance genes sta, novA, macB, tetA, vanS, adeF, and rphA. Our strain was closely related to other strains, and they formed a clade with humans and seawater isolates from Germany, the USA, China, and France (Fig. 1).

**TABLE 1:** Complete genome sequence information of *Kytococcus sedentarius* isolated from fish species

Bacterial isolate	*Kytococcus sedentarius* IFKSLREK1
Sequencing method	PacBio sequel II
HiFi reads	311,737,818
Length of contig	2,598,617
No. of contigs	1
Total length (bp)	2,598,617
*N*_50_	2,598,617
GC%	71.7
Contamination	–
Sequencing coverage	119.96
Complete BUSCOs (C)	97.6
Complete and single-copy BUSCO (S)	94.4
Complete and duplicated BUSCOs (D)	0
Fragmented BUSCOs (F)	2.4
Missing BUSCOs (M)	0
No. of coding sequences	2,393
No. of rRNAs	6
No. of tRNAs	51
No. of tmRNAs	1
Antimicrobial resistance genes (total)	2,173
BioProject number	PRJNA1152948
BioSample accession no.	SAMN43381806
Accession number	CP169391

## Data Availability

The whole-genome sequence of IFKSLREK1 is available in GenBank under accession number CP169391. The BioSample and BioProject accession numbers are SAMN43381806 and PRJNA1152948, respectively. The raw sequence data have been deposited in the Sequence Read Archive under accession number SRR30907435.
